# Urinary cytokines in women with refractory detrusor overactivity: A longitudinal study of rotating antibiotic versus placebo treatment

**DOI:** 10.1371/journal.pone.0247861

**Published:** 2021-03-03

**Authors:** Zhuoran Chen, Samantha Ognenovska, Ronald Sluyter, Kate H. Moore, Kylie J. Mansfield

**Affiliations:** 1 Department of Urogynaecology, St George Hospital, University of New South Wales, Sydney, New South Wales, Australia; 2 School of Chemistry and Molecular Bioscience, University of Wollongong, Wollongong, New South Wales, Australia; 3 Illawarra Health and Medical Research Institute, Wollongong, New South Wales, Australia; 4 School of Medicine, University of Wollongong, Wollongong, New South Wales, Australia; Northwestern University Feinberg School of Medicine, UNITED STATES

## Abstract

Over 50% of women with detrusor overactivity (DO), who do not respond to therapy have been shown to have bacteriuria, which may stimulate the release of inflammatory cytokines than can enhance nerve signalling, leading to symptoms of urgency. This study made use of a consecutive series of urine samples collected from women with refractory DO, who participated in a clinical trial of rotating antibiotic therapy. The aim was to determine the effect of bacteriuria and antibiotic treatment on the levels of urinary cytokines, and to correlate the cytokine concentration with patient outcome measures relating to urgency or urge incontinence. The urinary cytokines chosen were IL-1α, IL-1 receptor antagonist, IL-4, IL-6, IL-8, IL-10, CXCL10 (IP-10), MCP-1 and TNF-α. The presence of bacteriuria stimulated a significant increase in the concentrations of IL-1α (P 0.0216), IL-1 receptor antagonist (P 0.0264), IL-6 (P 0.0003), IL-8 (P 0.0043) and CXCL-10 (P 0.009). Antibiotic treatment significantly attenuated the release of IL-1α (P 0.005), IL-6 (P 0.0027), IL-8 (P 0.0001), IL-10 (P 0.049), and CXCL-10 (P 0.042), i.e. the response to the presence of bacteria was less in the antibiotic treated patients. Across the 26 weeks of the trial, antibiotic treatment reduced the concentration of five of the nine cytokines measured (IL-1α, IL-6, IL-8, IL-10 and CXCL-10); this did not reach significance at every time point. In antibiotic treated patients, the urinary concentration of CXCL-10 correlated positively with four of the six measures of urgency. This study has shown that cytokines associated with activation of the innate immune system (e.g. cytokines chemotactic for or activators of macrophages and neutrophils) are reduced by antibiotic therapy in women with refractory DO. Antibiotic therapy is also associated with symptom improvement in these women, therefore the inflammatory response may have a role in the aetiology of refractory DO.

## Introduction

Previous small studies have shown elevated levels of inflammatory cytokines in patients with urge incontinence and overactive bladder syndrome (OAB) [1–6]. One stimulus for the release of inflammatory cytokines is the presence of bacteria within the urinary tract [3, 7–9]. Release of IL-6 and IL-8 have been considered markers for urinary tract infection [3, 7, 9], with the presence of these cytokines correlating with symptomatic urinary tract infection (UTI) in elderly patients [9]. Uropathogens commonly associated with bacteriuria, such as *Escherichia coli* and *Enterococcus faecalis*, are capable of adherence to superficial urothelial cells and forming intracellular bacterial communities (IBCs) [10, 11]. IBCs represent an intracellular niche from which bacteria can trigger recurrent infections [12], leading to an enhanced immune response [13] and increased cytokine production [14]. IBCs have been demonstrated in urothelial cells exfoliated from women and children with acute UTI and with urge incontinence [12, 15–17].

As regards women with urge incontinence, in the last 10 years there has been increasing recognition that one third of patients respond poorly to the traditional first line therapy, namely anticholinergic medications [18]. These patients are termed refractory. Amongst such refractory patients, bacterial cystitis has been demonstrated to occur in 30–60% of cases [19–21]. More recently, three research groups have proposed that the urinary microbiome is altered in women with urge incontinence, and that these alterations are more pronounced in patients with the refractory form of the condition [22]. Although the pathophysiology of refractory urge incontinence remains incompletely understood, evidence is mounting to support the concept that in the subset of such refractory women with proven recurrent bacterial cystitis, the associated inflammatory response may be aetiological.

Women with urge incontinence and OAB experience debilitating urinary urgency, that is a strong desire to void for fear of leakage [23] associated with frequency and nocturia, with or without urge incontinence. In women with urge incontinence, urodynamic testing reveals Detrusor Overactivity (DO), where uncontrolled detrusor contractions occur during episodes of urgency (urge incontinence). Our hypothesis is that the enhanced inflammatory response, in women with recurrent bacteriuria and urge incontinence sensitises the suburothelial afferent nerves, leading to enhanced nerve signalling and greater urgency [24–26]. This may contribute to the poor response to anticholinergic therapy and thus the refractory state.

This possible aetiological link between bacteriuria and urge incontinence has triggered two open prospective trials of rotating antibiotic therapy for patients with refractory urge incontinence, but no placebo group was included [27, 28]. Both demonstrated improvements in urinary leakage and urgency symptoms, but cytokine responses were not studied. Recently, a randomised placebo controlled trial (RCT) of antibiotics in patients with refractory DO has demonstrated a significant improvement in patient symptoms, including a decrease in leakage (on 24-hour pad test) and voids per day (on bladder diary) in the antibiotic treated patients, and improvements in scores related to urinary urgency and symptom bother [29]. The response of cytokine levels to antibiotic therapy has not been studied.

The aims of the current study were to make use of the consecutive series of urine samples collected from women with refractory DO who participated in a RCT of rotating antibiotic therapy [29], and to determine the effect of bacteriuria, and antibiotic treatment, on the levels of urinary cytokines in such women. The urinary cytokines chosen were IL-1α, IL-1 receptor antagonist, IL-4, IL-6, IL-8, IL-10, CXCL10 (IP-10), MCP-1 and TNF-α. These cytokines have been shown in previous studies to be altered in women with urge incontinence [1–3, 5, 6], or to be important to the response to bacteriuria [1, 3, 7–9, 14]. Previous studies of urinary cytokines in women with OAB have generally focused on single-point data collection, in both urine and serum, comparing the results from those with urge incontinence to healthy controls. By making use of longitudinal samples collected over the six month period of the RCT we were able to examine the changes in cytokine levels over time, and to correlate the concentration of urinary cytokines with patient outcome measures relating to urgency or urge incontinence symptoms.

## Methods

### Participants

Patients and urine samples for this study came from the women who participated a double-blind, placebo-controlled, randomised clinical trial [29]. Selection criteria included postmenopausal status and >50 years of age with symptoms of overactive bladder (severe urgency, frequency, nocturia). Participants also had refractory urge incontinence (defined as persistent symptoms despite lifestyle modification, bladder training and two anticholinergics for more than one year). All participants had urodynamically proven DO without neurogenic DO, renal or voiding dysfunction. All participants provided written informed consent and the protocol was approved by the Ethics review committee (protocol CT13/003 Wollongong; HREC14/193 South East Sydney; registered with the Australia and New Zealand Clinical Trials Registry (ACTRN12613000285752)). Based on Simons two-stage design, to detect a biological effect of antibiotic therapy on urinary leakage, for an 80% power with 95% confidence, 120 trial participants were needed with a planned interim analysis after 33 enrolled patients [29]. Participants were randomised via minimization method at a 2:1 ratio of antibiotic therapy to placebo.

Prior to trial recruitment, all patients were pre-screened for any current classical symptoms of bacterial cystitis, and any symptomatic UTI was treated with an appropriate antibiotic (Trimethoprim or Cephalexin) prior to randomisation. Those randomised to the antibiotic group received two weeks each of Norfloxacin (400 mg, twice daily), Augmentin Duo (amoxycillin 500 mg/clavulanic acid 125 mg, twice daily) and Nitrofurantoin (100 mg, four times per day) sequentially. These antibiotics were chosen because all are commonly used to treat UTI [30, 31]. In those randomised to the placebo group, identical placebo tablets were given. All women received Darifenacin (15 mg daily), a highly bladder selective anticholinergic, for the entire six-month study period.

Regardless of trial allocation, any patients who experienced disabling symptoms of classical UTI were treated as per “clinical override”, which entailed administration of antibiotics (either Trimethoprim or Cephalexin).

### Sample collection

Midstream urine (MSU) samples were collected at randomisation (week 0), at weeks 2, 4, and 6 (during antibiotic/ placebo treatment), at week 10 (one month after the conclusion of the active treatment period), and week 26 (five months follow up after the active treatment period, the end of the six-month trial). Thus, a total of six MSU samples were collected per patient. Half of each MSU sample was sent to the hospital Microbiology department for routine culture. Bacterial cystitis (UTI) was determined by the presence of a single bacterial species (>10^5^ colony forming units (CFU)/L), usually with pyuria >10 white blood cells/high power field [32]. Polymicrobial bacteriuria was defined as more than one bacterial species isolated, with or without epithelial cells, with or without pyuria. Polymicrobial bacteriuria was included in this study as culture independent methods have indicated that mixed growth of organisms may be a precursor to later classical UTI in refractory DO [33]. For the purposes of this study, culture results of UTI and polymicrobial infection were combined into one group of ‘bacteriuria’. The remaining half of the urine sample was centrifuged (160*g* for 10 min) to remove exfoliated urothelial cells before being stored at -80°C.

### Cytokine analysis

Nine cytokines were measured using ELISA kits. Kits for IL-1α, IL-6, IL-8, IL-10, CXCL10 (IP-10) and TNF-α were from R&D Systems (Minneapolis, USA), with the remaining kits (IL-1 receptor antagonist, IL-4 and MCP-1) from Sigma-Aldrich (St Louis, USA). All cytokine analyses were performed as directed by the manufacturer. Urinary cytokine concentrations were compared between antibiotic and placebo groups (Mann-Whitney test), and also between bacteriuria (UTI and polymicrobial combined) and sterile results on MSU (one-way ANOVA with Dunns multiple comparisons). When comparing the effects of bacteriuria on urine cytokine concentrations, samples with bacteriuria were only included in the bacteriuria group on the day of the infection. Cytokine concentrations in the six urine samples per patient were also compared longitudinally between the antibiotic and placebo treatment groups at each individual time point (multiple t-test with Holm-Sidak correction for multiple comparisons). All statistical analyses were performed using Prism (Version 6).

### Clinical outcome measures

Several clinical outcome measures were collected as part of the RCT (described in Table 1) in order to determine the degree of urgency symptoms. These were subsequently correlated with the urinary cytokine concentrations (linear regression). As shown in Table 1, these measures were collected at randomisation (week 0), week 6 (at the end of the active treatment period), and at 6 months. These include two measures obtained from a 3-day bladder diary, voids per day (urinary frequency), and urgency severity based on Patient Perception of Intensity of Urgency Scale (PPIUS) [34]. The percentage of voids on the 3-day bladder diary scored as 3 (severe urgency) or 4 (urgency leading to incontinence) on the PPIUS were also calculated. Separate analysis of these higher grade scores has previously been suggested to be a better measure of urgency than analysis of the overall average PPIUS score [35]. Participants also completed the Overactive Bladder questionnaire (OABq) [36], which included eight questions relating to symptom severity (OABq SYS). The OABq SYS was further analysed by determining a symptom score for the subtotal of scores that particularly related to urgency (i.e. for questions 2, 3 and 7, Table 1). Two measures of urge incontinence were also collected as part of the RCT (Table 1). This included the 24-hour Pad Test, which was the primary outcome measure for the clinical trial. The 24-hour Pad Test was considered a measure of urge incontinence as all patients recruited to the trial had proven DO with minimal stress leak. The second measure of incontinence was leaks per day, determined from the 3-day bladder diary.

**Table 1:** Clinical outcome measures collected during the clinical trial.

**Test**	What it measures
***Measures of Urgency***	
**Voids per day**	Measure of urinary frequency, collected from a 3-day bladder diary
**PPIUS** ^a^	A measure of urgency where the patients rate their sensation for each void on a scale of 0 to 4 whereby:
	0 indicates no urgency
	1 indicates mild urgency
	2 indicates moderate urgency but can postpone for a while without fear of leakage
	3 indicates severe urgency, voiding is unable to be postponed
	4 indicates patient didn’t make it to the toilet, leaked (incontinence)
**% 3 or 4 PPIUS**	The percentage of voids that were classified as being associated with severe urgency
**OABq SYS** ^b^	Eight questions from the OABq including a measure of overactive bladder symptom severity incontinence. Patients report on the degree of symptom bother using a scale from 1 to 6 whereby 1 indicates no bother and 6 indicates a very great deal of bother
**OABq Urgency score**	Derived from the Urgency questions in OABq SYS, of the following symptoms questions:
	During the past 4 weeks, how bothered were you by:
	Q2 an uncomfortable urge to urinate
	Q3 a sudden urge to urinate with little or no warning
	Q7 an uncontrollable urge to urinate
***Measures of Incontinence***	
**Pad Test**	Weight of urine leakage in a 24-hour period, in grams on pre-weighed pads
**Leaks per day**	Number of incontinence episodes per day, collected from a 3-day bladder diary

^a^ PPIUS Patient Perception of Intensity of Urgency Scale.

^b^ OABq SYS Overactive Bladder Questionnaire symptom severity.

## Results

### Effect of bacteriuria on cytokine release

All 36 patients recruited to this trial were randomised according to the study protocol. Patients randomised to the antibiotic and placebo groups were evenly matched in terms of age and history of recurrent UTI (Table 2). At randomisation, there was no difference in the proportion of women who were found to have bacteriuria (P = 0.711, Table 2). As would be expected during the active treatment period (weeks 2, 4 and 6), women randomised to the antibiotic group were more likely to have ‘no growth’ on MSU, while those in the placebo group were more likely to have bacteriuria (P = 0.0041, Table 2). During the 5-month post-treatment longitudinal follow-up, women who had received antibiotic therapy remained more likely to have ‘no growth’ on MSU than those in the placebo group (P = 0.006, Table 2).

**Table 2:** Patient characteristics and presence of bacteriuria.

	Placebo group (n = 12)	Antibiotic group (n = 24)	Chi-square p-value
Age in years, Mean (SD)	67.1 (7.9)	67.6 (8)	
History of recurrent UTI	25% (3/12)	33% (8/24)	
MSU result at randomisation			
No growth	33% (4/12)	27% (6/22)	0.71
Bacteriuria	67% (8/12)	73% (16/22)	
MSU result during the treatment period			
No growth	22% (8/36)	52% (34/66)	**0.004**
Bacteriuria	78% (28/36)	48% (32/66)	
MSU result during the post-treatment, follow-up period			
No growth	0%	26% (11/42)	**0.006**
Bacteriuria	100% (24/24)	74% (31/42)	

Urinary cytokine concentrations of no growth and bacteriuria urine samples were compared within each treatment group, as well as between placebo and antibiotic treated patients (Table 3). These comparisons included all samples collected across the 6-months of the trial. In patients with no growth, there was a significant increase in IL-6 (P = 0.045) in the antibiotic treated women. As would be expected, in the placebo patients, the presence of bacteriuria stimulated a significant increase in the urinary concentrations of various cytokines including IL-1α (P = 0.0216), IL-1 receptor antagonist (P = 0.0264), IL-6 (P = 0.0003), IL-8 (P = 0.0043) and CXCL-10 (P = 0.009) (Table 3). A similar but lesser cytokine response was observed in the antibiotic treated patients, with only IL-1α (P = 0.0016), IL-1 receptor antagonist (P = 0.0203) and IL-4 (P = 0.023) significantly increasing in response to bacteriuria.

**Table 3:** Urinary cytokine concentrations of placebo and antibiotic treated women with refractory DO, with no growth or with bacteriuria.

	No Growth		Bacteriuria	
	Placebo	Antibiotic	Placebo	Antibiotic
IL-1α	3.37 (2.3–4.9; 11)	1.51 (0.65–3.4; 51)	6.5^#^ (3.2–15.4; 52)	2.82* (1.5–5.9; 81)
IL-1 receptor antagonist	1.03 (0.2–2.1; 11)	0.91 (0.4–2.2; 51)	2.13^#^ (0.8–6.9; 52)	1.37* (0.6–3.8; 81)
IL-4	0.62 (0.3–1.1; 11)	0.65 (0.3–2.2; 51)	0.66 (0.4–1.1; 52)	1.65* (0.6–1.5; 81)
IL-6	0.87 (0.4–1.8; 11)	1.64^$^ (1.0–2.5; 51)	2.91^#^ (1.5–7.15; 52)	1.83 (1.1–3.7; 81)
IL-8	9.18 (4.8–29.6; 11)	15.61 (8.1–34.8; 51)	45.07^#^ (24.5–187; 52)	16.8 (11.0–45.6; 81)
IL-10	10.06 (3.2–12.6; 7)	6.53 (1.9–11.0; 30)	6.17 (3.5–10.3; 30)	4.07 (2.3–8.0; 50)
CXCL-10	5.01 (3.1–11.9; 11)	9.17 (2.8–24.1; 51)	16.86^#^ (6.6–43.4; 51)	9.87 (3.5–28.7; 81)
MCP-1	6.64 (3.4–30.4; 11)	9.53 (3.6–24.1; 51)	9.13 (4.3–24.6; 51)	11.04 (2.5–34.5; 81)
TNF-α	0.11 (0.08–0.2; 8)	0.083 (0.05–0.1; 39)	0.095 (0.07–0.2; 37)	0.095 (0.06–0.12; 69)

Cytokine concentrations are shown as median (IQR; n).

^$^ significant difference in cytokine release between placebo and antibiotic in respective no growth group

^#^ significant increase in cytokine release in response to bacteriuria (compared to no growth) in the placebo group

* significant increase in cytokine release in response to bacteriuria (compared to no growth) in the antibiotic group

**Fig 1:**
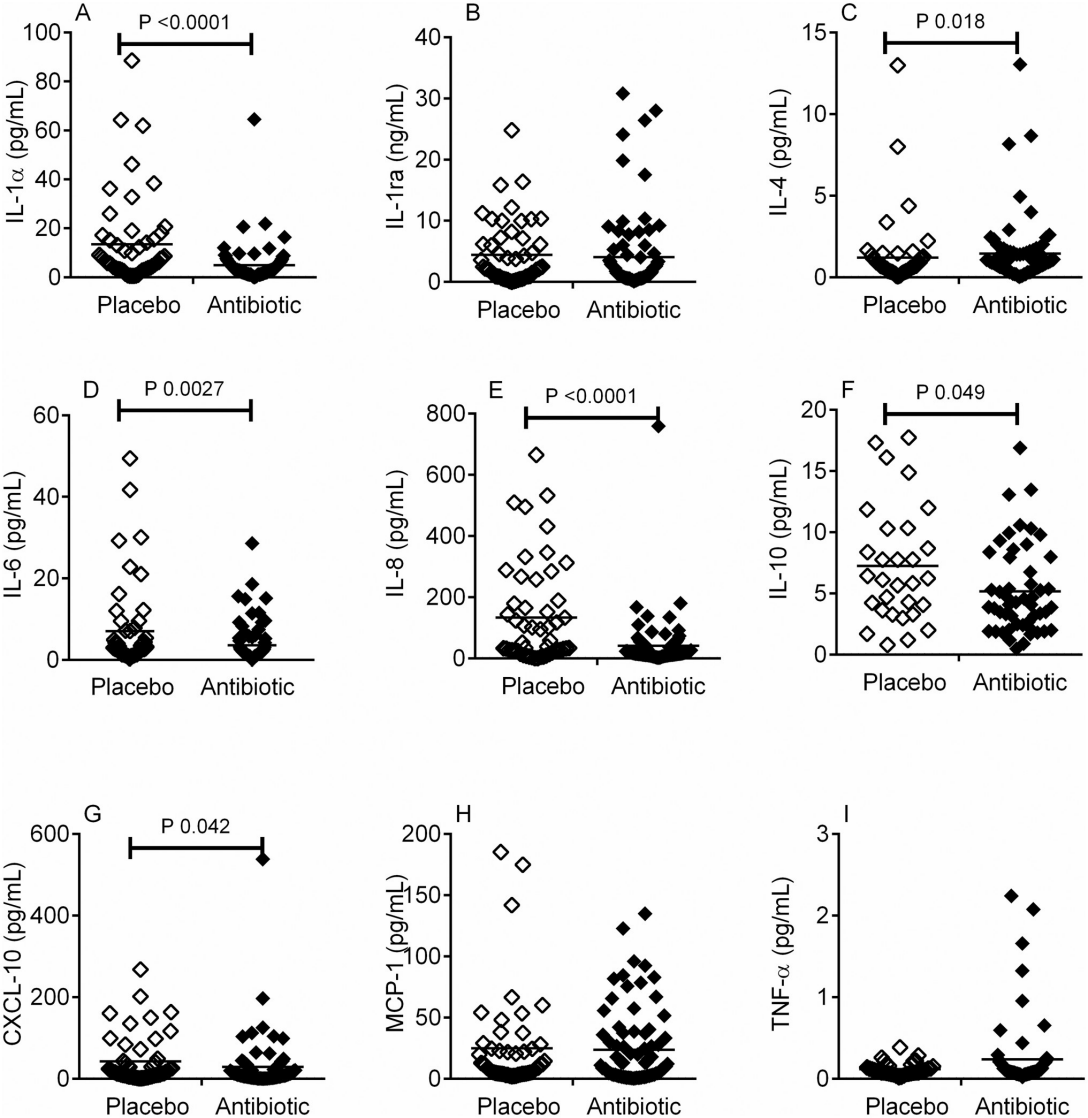
In patients with bacteriuria, concentration of urinary cytokines in placebo (◇) and antibiotic (◆) treatment groups. The concentration of urinary cytokines IL-1α (A), IL-1 receptor antagonist (ra) (B), IL-4 (C), IL-6 (D), IL-8 (E), IL-10 (F), CXCL10 (IP-10) (G), MCP-1 (H) and TNF-α (I) were measured by ELISA in women with bacteriuria (both UTI and polymicrobial infections) in both the antibiotic and placebo treatment groups. The cytokines IL-1α (A), IL-6 (D), IL-8 (E), IL-10 (F) and CXCL10 (G) were significantly lowered in the antibiotic treatment group in response to the presence of bacteriuria, compared placebo. In contrast, IL-4 (C) was significantly increased in the antibiotic treatment group compared to placebo. Data analysed using Mann-Whitney test and with the horizontal line across each column representing the median.

In the antibiotic treated patients, who developed bacteriuria, the antibiotic treatment significantly attenuated the cytokine response to bacterial presence (i.e. comparing cytokine responses to bacteriuria between placebo and antibiotic treated patients, Table 3). This was observed as relative reductions in the concentrations of IL-1α (Fig 1A, P = 0.005), IL-6 (Fig 1D, P = 0.0027), IL-8 (Fig 1E, P < 0.0001), IL-10 (Fig 1F, P = 0.049), and CXCL-10 (Fig 1G, P = 0.042) in patients with bacteriuria. In contrast the concentration of IL-4 was significantly increased in the antibiotic patients with bacteriuria compared to that detected in patients with bacteriuria in the placebo group (Fig 1C, P = 0.018).

### Effect of antibiotic treatment on cytokine release

**Fig 2:**
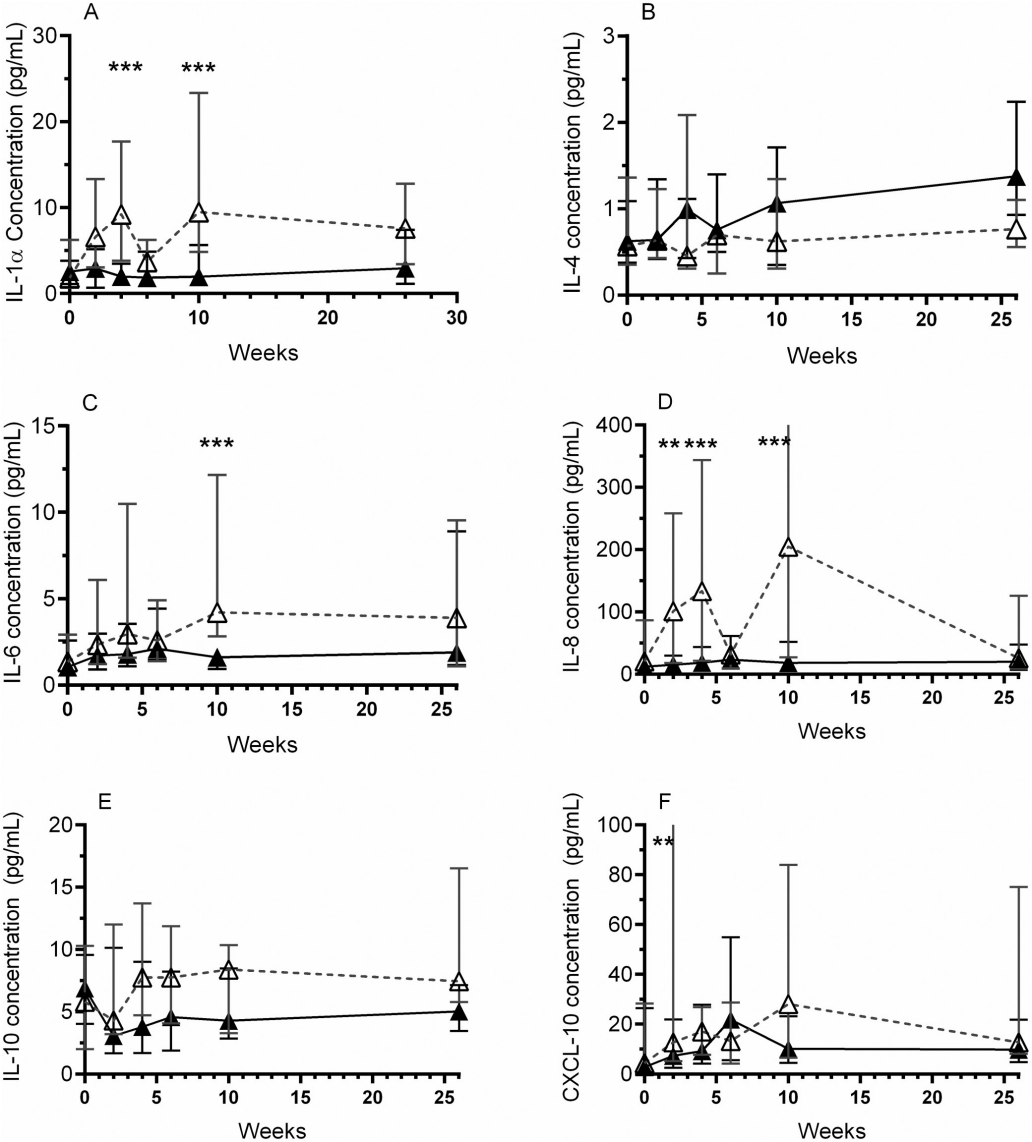
Longitudinal effect of antibiotics on urine cytokine concentrations. The concentration of urinary cytokines were measured by ELISA and compared over time in the antibiotic (▲) and placebo (△) treatment groups (regardless of bacterial culture result). The concentrations of IL-1α (A), IL-6 (C) and IL-8 (D) were consistently significantly less in the antibiotic treatment group, compared to placebo. The concentration of IL-10 (E) was lower in the antibiotic treatment group than placebo, although this did not reach significance. The comparisons between the concentration of CXCL-10 (F) in the antibiotic and placebo treatment groups over time was more variable. Analysis was performed using a Holm-Sidak multiple comparisons t-test, where: ** = p<0.01, *** = p<0.001. Data presented as median and IQR.

Next, a group wise longitudinal comparison of cytokines in samples collected from all antibiotic treated women (regardless of bacterial culture result) was compared with samples collected from all placebo treated women (regardless of bacterial culture result) at each time point. The concentration of IL-1α, IL-4, IL-6, IL-8, IL-10 and CXCL-10 was compared longitudinally over the entire 26 weeks of the trial (Fig 2). At each time point tested the antibiotic treated patients exhibited lower concentrations in five of the nine urinary cytokines measured, compared to the placebo (Fig 2). However, this did not reach significance at every time point (multiple t-test with Holm-Sidak correction for multiple comparisons).

During the active treatment (weeks 2, 4 and 6), there was a significant decrease in the concentration of IL-1α (Fig 2A, 4 weeks P 0.0013), IL-8 (Fig 2D, 2 weeks P 0.0022; 4 weeks P < 0.0001) and CXCL-10 (Fig 2F, 2 weeks P 0.0076). At 10 weeks (one month after the conclusion of the active treatment), the concentration of IL-1α (P 0.0035), IL-6 (P 0.001), and IL-8 (P < 0.0001) remained lower in the antibiotic group compared to placebo. Interestingly, similar to the results in Table 3, IL-4 was seen to be higher in the antibiotic treated patients at almost every time point measured (Fig 2B). However, this did not reach significance.

### Correlation of cytokine concentrations with clinical outcome measures

To determine the relationship between cytokine release and patient symptom severity, the concentrations of urinary cytokines were correlated with the clinical outcome measures collected as part of the RCT (described in Table 1). When data from all patients was considered together (n = 34 patients; n = 98 MSU specimens, Table 4) the urinary concentration of CXCL-10 positively correlated with OABq symptoms score (P = 0.031), indicating that higher concentrations of CXCL-10 correlated with increasing symptom bother. In addition, urinary concentrations of TNF-α negatively correlated with PPIUS (P = 0.035), demonstrating that higher concentrations of TNF-α correlated with lower urgency.

**Table 4:** Correlations between cytokine concentrations and clinical outcome measures.

	All patients (n = 34 patients; 98 urines)	Antibiotic (n = 24 patients; 63 urines)
*Measures of Urgency*		
Voids/ day		IL-1 ra* (slope = -0.67, r^2^ = 0.068, P = 0.039)
PPIUS^a^	TNF-α (P = 0.035, slope = -0.069, r^2^ = 0.058)	CXCL-10 (slope = 26.54, r^2^ = 0.087 P = 0.019)
% 3 or 4 PPIUS		CXCL-10 (slope 0.82, r2 0.097, P 0.016)
OABq SYS^b^	CXCL10 (P = 0.031, slope = 0.63, r^2^ = 0.048)	IL-6 (slope = 0.063, r^2^ = 0.078, P = 0.026)
		CXCL-10 (slope = 1.18, r^2^ = 0.13, P = 0.034)
OABq Urgency score		CXCL-10 (slope = 5.06, r^2^ = 0.07, P = 0.036)
*Measures of incontinence*		
Pad weight		IL-1α (slope = 0.013, r^2^ = 0.11, P = 0.0071)

* ra receptor antagonist.

^a^ PPIUS Patient Perception of Intensity of Urgency Scale.

^b^ OABq SYS Overactive Bladder Questionnaire symptom severity.

When correlations were assessed using data from antibiotic treated patients alone (n = 24 patients; n = 63 MSU specimens), a number of significant correlations were observed with clinical measures of urgency. The urinary concentration of CXCL-10 was observed to correlate with four of the six measures of urgency (Table 4). Urinary CXCL-10 levels significantly increased with both the average PPIUS score (P = 0.019) and the percentage of voids scored 3/4 on the PPIUS scale (P = 0.0015). In addition, OABq SYS (P 0.034) and OABq SYS Urgency score (P = 0.036) were found to increase as CXCL-10 concentration increased. Similarly, the concentration of IL-6 also positively correlated with OABq SYS (P = 0.0257).

In relation to measures of incontinence, the concentration of IL-1α was found to significantly increase with pad weight (P = 0.007, Table 4), while IL-1 receptor antagonist concentration decreased as the average frequency of voids per day increased (P 0.039).

## Discussion

To our knowledge this is the first longitudinal study that analyses successive urinary cytokine concentrations over 26 weeks, which is pertinent because the symptoms of urge incontinence are known to wax and wane over time [37]. The longitudinal nature of this study allowed correlation between the cytokine concentrations and the presence/ absence of bacteriuria, and between cytokine levels and patients’ symptoms. All previous studies have only included a single time point. Also, this study recruited only women with urodynamically proven refractory DO. In contrast, most previous cytokine studies recruited patients with “overactive bladder” symptoms (OAB) [1–6, 38, 39], a clinical syndrome including urgency and frequency with or without incontinence, who were not refractory [23]. As such, all of our subjects had a precisely defined physiological abnormality.

In this cohort of women with refractory DO, a history of recurrent UTI was noted in one third of participants and bacteriuria was found at randomisation in two thirds (24/36) of study participants. This accords with previous reports that up to 50% of women with refractory DO have bacteriuria [19–21, 33, 40–43]. In patients given placebo, bacteriuria triggered an elevation in urinary cytokine levels for IL-1α, IL-1 receptor antagonist, IL-6, IL-8 and CXCL-10.

IL-1α is part of the normal cytokine response to bacterial endotoxins, and has been shown to be elevated in UTI [7]. Cytokines, IL-6 and IL-8 activate phagocytic immune cells (macrophages and neutrophils, respectively). IL-6 and IL-8 are released in response to UTI [1, 3, 7, 9]. IL-6 is produced by bladder urothelial cells in response to infection [7, 13, 14]; elevation in IL-6 has been reported in women with OAB [1]. CXCL10 (or IP10) is released in response to bacterial lipopolysaccharide and in response to IFN-ɣ (one of the first cytokines released in the presence of bacteria). CXCL10 is chemotactic for macrophages and thus enhances the innate immune response to infection, and is part of the inflammatory response to injury. Increased levels of CXCL10 have previously been reported in women with UTI [3] but not in women with urge incontinence [3, 39]. These findings highlight the role of the innate immune system in the bladder response to bacteriuria.

In contrast to the above cytokines (IL-6, IL-8, CXCL-10), the IL-1 receptor antagonist is an anti-inflammatory cytokine that functions to block the actions of IL-1α and IL-1β [44]. Previous studies of OAB have reported a decrease in the level of IL-1 receptor antagonist [3]. High levels of IL-1 receptor antagonist are thought to be prophylactic against UTI [3]. Therefore, the increased levels of IL-1 receptor antagonist in women with bacteriuria may be a protective response against IL-1α release in the bladder.

The present results show that antibiotic therapy reduced cytokines release in response to bacteriuria. This is despite one of the limitations of the study, i.e. small sample size of the RCT. The trial was halted post interim analysis because of low recruitment (due mainly to the arrival of a new treatment, botulinum toxin injections that was highly attractive to patients) and also because of ethical concerns that many patients on placebo needed antibiotic therapy (“clinical override”, for details see ref 29). Nevertheless, a clinical benefit was seen at the pre-designed interim analysis. Furthermore, urine collection yielded 202 samples, giving a sample size for the cytokine data that was more than adequate, based on one previous study [3].

In antibiotic treated patients, five of the nine cytokines (IL-1α, IL-6, IL-8, IL-10 and CXCL10) were significantly reduced in the presence of bacteriuria (compared to the cytokine response in those on placebo). As mentioned, four of these cytokines (IL-1α, IL-6, IL-8 and CXCL10) are associated with activation of the innate immune response to infection. CXCL10 was also shown to positively correlate with four of the six clinical measures of urgency; with an increasing concentration of CXCL-10 associated with higher urgency scores. We therefore conclude that antibiotic therapy not only decreases the incidence of bacteriuria in refractory DO, but also decreases the stimulus for activation of the innate immune response (e.g. activation of macrophages, phagocytosis of bacteria) and decreases the associated tissue damage. This reduction in tissue damage following antibiotic therapy would likely lead to reduced activation of afferent nerves and subsequently reduced urgency. Another limitation to our study is that urinary cytokines may not reflect the concentration of cytokines in the suburothelial space. Ideally, concurrent bladder biopsies could provide this correlation, however due to the invasive nature of biopsies, this was not ethically feasible in this RCT

In addition to decreasing the concentration of these pro-inflammatory cytokines, antibiotic treatment also decreased the concentration of the anti-inflammatory cytokine IL-10, in response to bacteriuria. IL-10 typically inhibits the release of inflammatory cytokines in response to bacterial lipopolysaccharide [45]. Previous studies of urinary IL-10 have shown variable results; some studies report increased IL-10 in OAB [2], and also in elderly patients with asymptomatic bacteriuria [9], while others report decreased IL-10 in OAB [39]. The only other well-described anti-inflammatory cytokine in our study was IL-4, which has previously been shown to be decreased [5] or unchanged [39] in OAB patients. However, in animal UTI models, IL-4 appears to protect against bladder inflammation [5]. In the current study, IL-4 was elevated in antibiotic-treated patients with bacteriuria. Whether this increase reflects immune suppression or immune deviation, such as alternative activation of macrophages [46], remains currently unknown.

The RCT of antibiotics in women with refractory DO demonstrated improvement in urge incontinence [29]. Not only were the measures of urgency (OABq SYS and voids per day) significantly reduced in women on antibiotics, they also showed a substantial reduction in urge incontinence on the primary outcome measure (24 hour pad test). Understanding the physiological mechanism by which antibiotics lead to clinical improvement in refractory DO is important, particularly in this era of antibiotic stewardship, as increasing antibiotic use in the community is contributing to rising incidence of multi-drug- resistant organisms [47]. The current study has shown that antibiotics reduce cytokine release, and that the concentrations of urinary cytokines correlate with measures of urgency/ urge incontinence. This suggests the clinical improvement following antibiotic therapy is likely to result from the suppression of the inflammatory response.

This opens up potential new treatment options. For example, two anti-inflammatory agents Diclofenac [48] and Ibuprofen [49] have previously shown benefit for patients with acute UTI. Similarly, older studies of indomethacin showed benefit for OAB symptoms [50–52]. More recently, the therapeutic properties of agents specifically targeting individual cytokines have been investigated. Blockade of receptors activated by CXCL10 has been shown to increase bladder capacity, reduce bladder contractility and nerve sensitivity in a murine model of interstitial cystitis [53]. Antibodies developed to block TNF have revolutionised the treatment of inflammatory bowel diseases, Crohn’s disease and ulcerative colitis [54].

In conclusion, our study demonstrates that antibiotics not only reduce the incidence of bacteriuria in refractory DO but also persistently decrease the inflammatory cytokine response over time. This prolonged reduction in pro-inflammatory cytokines is likely to be associated with suppression of the innate immune response, lessened tissue damage and therefore decreased activation of afferent nerves with reduced urgency. These novel findings shed new light on our understanding of the aetiology of refractory DO, in women who have persistent bacteriuria and do not respond to current anticholinergic therapy.

## Supporting information

S1 Appendix (XLSX)
